# Who’s Missing Out? Exploring Potential Medicaid Enrollment Gaps among People with Dementia

**DOI:** 10.1093/geroni/igaf122.729

**Published:** 2025-12-31

**Authors:** Sijiu Wang, Siying Yang, Jing Li

**Affiliations:** Tsinghua University, Beijing, Beijing, China (People’s Republic); Tsinghua University, Beijing, Beijing, China (People’s Republic); University of Washington, Seattle, Washington, United States

## Abstract

Medicaid serves as a critical safety net for older adults with Alzheimer’s Disease and Related Dementias (ADRD), yet many eligible individuals remain unenrolled, exacerbating their health and financial vulnerabilities. This study analyzes data from the National Health and Aging Trends Study (2011-2023), a nationally representative panel survey of Medicare beneficiaries, to identify Medicaid-eligible but unenrolled older adults with ADRD and assess their health and economic risks. We conducted a panel data analysis using survey waves immediately before and after Medicaid enrollment to examine enrollee characteristics, including health, functioning, and financial status prior to enrollment. Two approaches were employed to identify potentially eligible but unenrolled people with ADRD: (1) defining potential eligibility based on income below 100% and 138% of the Federal Poverty Level, and (2) estimating expected Medicaid enrollment likelihood using socioeconomic and health characteristics of non-ADRD enrollees as a reference. Comparative analyses revealed that unenrolled ADRD individuals were disproportionately aged 85+, Hispanic, and residing in the West, with higher rates of functional impairment, hospitalizations, and depressive symptoms compared to their enrolled counterparts. While they exhibited relatively better financial status than Medicaid enrollees with ADRD, their economic hardship was significantly greater than that of non-ADRD individuals. Findings suggest that structural barriers, rather than eligibility criteria alone, contribute to Medicaid enrollment gaps. Targeted outreach strategies, streamlined enrollment processes, and culturally responsive interventions are needed to close these gaps and mitigate adverse health and financial outcomes among this vulnerable population.

